# Abnormal cervical cytology amongst women infected with human immunodeficiency virus in Limpopo province, South Africa

**DOI:** 10.4102/phcfm.v12i1.2215

**Published:** 2020-10-06

**Authors:** Samuel T. Ntuli, Eric Maimela, Linda Skaal, Mabina Mogale, Provia Lekota

**Affiliations:** 1 Department of Public Health, Faculty of Health Sciences, University of Limpopo, Sovenga, South Africa; 2 Department of Public Health, Faculty of Health Sciences, Sefako Makgatho Health Sciences University, Ga-Rankuwa, South Africa; 3 Department of Statistics and Operations Research, Sefako Makgatho Health Science University, Ga-rankuwa, South Africa

**Keywords:** cervical cancer, human immunodeficiency virus, Limpopo province, Prevalence, abnormal cytology

## Abstract

**Background:**

Cervical cancer remains the major public problem worldwide and the most common gynaecological malignancy in the developing world, particularly in sub-Saharan Africa.

**Aim:**

To determine the prevalence of abnormal cervical cytology amongst women with and without human immunodeficiency virus (HIV) and examine the association between HIV and histological grading.

**Setting:**

The study was conducted in Limpopo province, which is the northernmost province of South Africa. The province has five district municipalities with one tertiary, five regional and thirty four district hospitals.

**Methods:**

We retrospectively reviewed cervical cancer cases in Limpopo province (LP) of South Africa, using data collected routinely by the National Health Laboratory Services (NHLS). The data on smears submitted for cytology between 2013 and 2015 were extracted from the Central Data Warehouse (CDW) database.

**Results:**

A total of 84 466 women were screened for cervical cytology smears. Their mean age was 39.8 ± 13.6 years, with range from 15 to 113 years; 77.2% were in the age group 30 years and older and 19.6% had an abnormal cervical cytology result. Overall, 46.4% of the women screened for cervical cancer were HIV infected. A significantly higher proportion of HIV-positive women had abnormal cytology than HIV-negative women (31.8% vs. 9.2%).

**Conclusion:**

The prevalence of abnormal cytology amongst HIV-positive women is relatively high, and the risk appears to be significantly greater in all age groups. This finding highlights the need to ascertain HIV status of all women presenting with cervical cancer.

## Introduction

Cervical cancer (CC) is the most common gynaecological malignancy in the developing world, particularly in sub-Saharan Africa.^1,2^ Prevention of complications and improved survival from CC depend on early detection, treatment and human papillomavirus (HPV) vaccination before any sexual debut.^3,4^ Human papillomavirus has been known to increase with age; however, some age groups remain at higher risk of CC, especially those living with human immunodeficiency virus (HIV). The prevalence of abnormal cytology amongst HIV-positive women in African countries is 5% – 30%.^5,6,7,8,9^ In KwaZulu-Natal province of South Africa (SA), 54.9% of women living with HIV had abnormal Papanicolaou (Pap) smear results.^10^ In Limpopo province (LP), 26.7% of the HIV-positive migrant farm workers and sex workers had abnormal cervical cytology tests.^11^ Not surprisingly, studies report significantly higher burdens of abnormal cervical cytology in HIV-positive women, compared with HIV-negative women.^12,13^ The national guidelines in SA clearly state that women aged 30 years or older should be offered three free lifetime Pap smears,^14^ with recommendations to screen HIV-positive women annually.^15^ Despite all this, most women do not receive adequate screening.^16,17,18^ In LP, the annual screening coverage rates increased from 2.9% in 2007 to 4.2% in 2010^19^; however, there is no information on the prevalence of cervical lesions amongst HIV-positive women. In an attempt to assess proportion of HIV-positive women with cervical lesions, our study analysed routinely collected data by the National Health Laboratory Services (NHLS).

## Methods

We retrospectively reviewed CC cases in LP using data collected routinely by the NHLS. The data on smears submitted for cytology between 01 January 2013 and 31 December 2015 were extracted from the Central Data Warehouse database. The variables used for this study include date of smear collection, the women’s ages at smear collection, HIV status, smear adequacy and smear cytology results. The details of the study setting, CC screening programme and data management are described in a previous study.^19^

Data were analysed using Stata® statistical software (Release 11; StataCorp, 2009, College Station, TX). Categorical and continuous variables were summarised using proportions (percentages) and means (standard deviation), respectively. The chi-square test was used to compare the two groups (i.e. HIV-positive and HIV-negative women). A *p-*value of less than 0.05 was considered to be statistically significant.

### Ethical consideration

Ethical approval was obtained from Turfloop Research Ethics Committee (Ref: TREC/219/2106; PG) and permission to use the data was obtained from the National Health Laboratory Services (Ref No.: 2019609).

## Results

**FIGURE 1:**
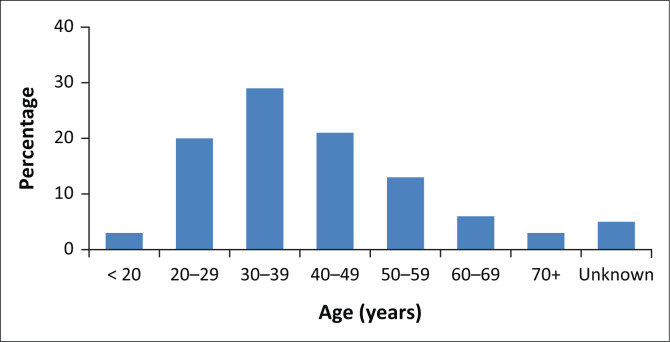
Distribution of age (years) for women screened for cervical cancer (*n* = 84 466).

**FIGURE 2:**
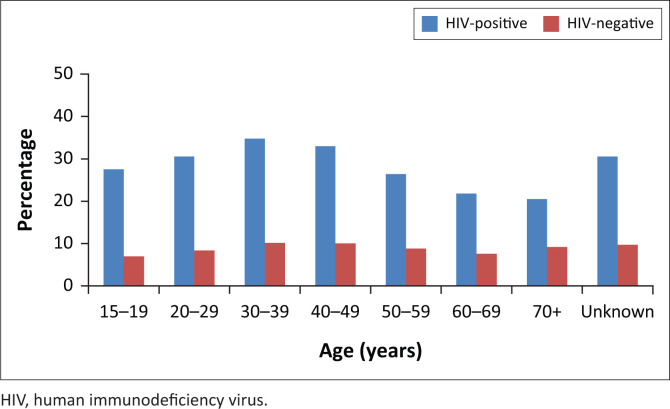
Distribution of abnormal cytology by age amongst human immunodeficiency--positive and human immunodeficiency-negative women.

A total of 84 466 women were screened for cervical cytology smears. Their mean age was 39.8 ± 13.6 years, with range from 15 to 113 years. Figure 1 shows the age distribution of the women who participated in this study. Overall, 46% of the women screened for CC were HIV-infected. As shown in Table 1, a higher proportion of HIV-infected women had abnormal cytology as compared with women without HIV infection (31.8% vs. 9.2%, *p* < 0.05). With regard to histological grading, overall, 78.6% was low-grade squamous epithelial lesions Cervical intraepithelial neoplasm (CINI) and 21.4% was high-grade squamous epithelial lesions (CIN II/III and carcinoma *in situ*). For comparison of histological grading between HIV-positive and HIV-negative women, a significant higher proportion of HIV-negative women had CIN II/III and carcinoma *in situ* than those who tested HIV positive (23.2% vs. 20.8%, *p* < 0.05). In all age groups, the prevalence of abnormal cytology was significantly higher amongst HIV-positive women than amongst women who tested HIV-negative (*p* < 0.05; Figure 2).

**TABLE 1:** Comparison of cytology results and histological grading between human immunodeficiency virus-positive and human immunodeficiency virus-negative women.

Cytology and histology	*N*	HIV-positive (%)		HIV-negative (%)		*p*
		*n*	%	*n*	%	
**Cytology result**						< 0.001
Normal	67 869	26 765	68.2	41 104	90.8	
Abnormal	16 597	12 455	31.8	4142	9.2	
**Histological grading**						< 0.001
CIN I	13 042	9864	79.2	3178	76.8	
CIN II/III and carcinoma *in situ*	3553	2591	20.8	962	23.2	

CIN I, atypical glandular cells of undetermined significance, atypical squamous cells of undetermined significance, low-grade intraepithelial lesion; CIN II, atypical squamous cells (cannot exclude high-grade squamous intraepithelial lesion, high-grade squamous intraepithelial lesion); CIN III, malignant and adenocarcinoma *in situ*; HIV, human immunodeficiency virus.

## Discussion

To our knowledge, this is the first study in LP to assess the prevalence of cervical lesions amongst HIV-positive women. Our finding shows that the prevalence of cervical lesions amongst HIV-positive women was 31.8%, which was significantly higher than 9.2% in HIV-negative women. This finding is slightly higher than 22% found in a study conducted in Swaziland^12^ but lower than 71.8% reported in Tanzania.^13^ Several studies conducted amongst women living with HIV found the prevalence of abnormal cytology of 54.9% in SA,^10^ 26.7% in Kenya,^5^ 26.8% in Tanzania,^6^ 22.1% in Ethiopia,^7^ 15.2% in Botswana^9^ and 6.0% in Nigeria.^8^ Afzal and co-authors in a study conducted in LP found that 26.7% of the HIV-positive migrant farm and sex workers had abnormal cytology.^11^ The reasons for various prevalence rates reported in these studies are unclear; however, it could be because of various methods used for CC screening^20,21,22,23^ and sexual practices of women,^7,8,12,24,25^ which is explained by higher burden of HPV infection.^26,27,28,29^

Despite the effectiveness of highly active retroviral therapy (ART) in preventing the development of cervical lesions,^7,8^ interestingly in our study, prevalence of abnormal cervical cytology was significantly higher in women with HIV than in women without HIV. Vafaei et al., in their study, reported a greater proportion of women with abnormal cervical cytology amongst HIV-positive women than in the general population.^30^ Although our study did not document CD4 count and/or whether HIV-positive women were on ART treatment or not, systematic reviews found that integration of cervical screening with HIV treatment is feasible and acceptable to women living with HIV.^31,32^

In accordance with SA national guideline on CC screening, our findings show that more than two-thirds of women screened were 30 years and older. Similarly, a study in Nigeria found that the majority of women screened for cervical lesions were 30 years and older.^8^ Not surprisingly, amongst younger women (< 30 years), abnormal cervical cytology was significantly higher in those with HIV. A retrospective study in Tygerberg Academic Hospital in Cape Town, SA, found many women younger than 30 years diagnosed with invasive CC.^33^ These findings support the recommendation made by Botha^33^ that all SA women should initiate cervical screening at the age of 25 years or at the time of diagnosis of HIV seropositivity.^34^

Studies in SA reported higher rates of CIN II/III and carcinoma *in situ* in HIV-positive women when compared with HIV-negative women.^35,36^ A similar finding was reported in LP amongst HIV-positive migrant farm workers and sex workers.^11^ In contrast, in this study, CIN II/III and carcinoma *in situ* were significantly higher amongst HIV-negative women than amongst HIV-positive women, which is in agreement with a study in Malawi.^37^ This study has several limitations similar to an earlier study in LP,^19^ which included limited clinical data such as missing HIV test results, unavailable CD4 count and ART treatment for HIV-positive women and screened patients not allocated the same laboratory identification number during follow-up visits.

## Conclusion

Our study shows that the prevalence of abnormal cervical cytology amongst HIV-positive women is relatively high and the risk appears to be significantly greater in all age groups particularly young women. This finding highlights the need to ascertain HIV status of all women presenting with CC and more importantly implementation of effective HPV vaccination in the prevention of opportunistic infections in HIV-positive adults and adolescents.^4^
